# Clinical Trials of the BTK Inhibitors Ibrutinib and Acalabrutinib in Human Diseases Beyond B Cell Malignancies

**DOI:** 10.3389/fonc.2021.737943

**Published:** 2021-10-28

**Authors:** Sining Zhu, Jaeyong Jung, Eton Victor, Johann Arceo, Samantha Gokhale, Ping Xie

**Affiliations:** ^1^ Department of Cell Biology and Neuroscience, Rutgers University, Piscataway, NJ, United States; ^2^ Graduate Program in Cellular and Molecular Pharmacology, Rutgers University, Piscataway, NJ, United States; ^3^ Rutgers Cancer Institute of New Jersey, New Brunswick, NJ, United States

**Keywords:** BTK, ibrutinib, acalabrutinib, immunomodulation, immune responses, inflammation, cancers, COVID-19

## Abstract

The BTK inhibitors ibrutinib and acalabrutinib are FDA-approved drugs for the treatment of B cell malignances. Both drugs have demonstrated clinical efficacy and safety profiles superior to chemoimmunotherapy regimens in patients with chronic lymphocytic leukemia. Mounting preclinical and clinical evidence indicates that both ibrutinib and acalabrutinib are versatile and have direct effects on many immune cell subsets as well as other cell types beyond B cells. The versatility and immunomodulatory effects of both drugs have been exploited to expand their therapeutic potential in a wide variety of human diseases. Over 470 clinical trials are currently registered at ClinicalTrials.gov to test the efficacy of ibrutinib or acalabrutinib not only in almost every type of B cell malignancies, but also in hematological malignancies of myeloid cells and T cells, solid tumors, chronic graft *versus* host disease (cGHVD), autoimmune diseases, allergy and COVID-19 (http:www.clinicaltrials.gov). In this review, we present brief discussions of the clinical trials and relevant key preclinical evidence of ibrutinib and acalabrutinib as monotherapies or as part of combination therapies for the treatment of human diseases beyond B cell malignancies. Adding to the proven efficacy of ibrutinib for cGVHD, preliminary results of clinical trials have shown promising efficacy of ibrutinib or acalabrutinib for certain T cell malignancies, allergies and severe COVID-19. However, both BTK inhibitors have no or limited efficacy for refractory or recurrent solid tumors. These clinical data together with additional pending results from ongoing trials will provide valuable information to guide the design and improvement of future trials, including optimization of combination regimens and dosing sequences as well as better patient stratification and more efficient delivery strategies. Such information will further advance the precise implementation of BTK inhibitors into the clinical toolbox for the treatment of different human diseases.

## Introduction

Bruton’s tyrosine kinase (BTK), a member of the TEC kinase family, was originally identified as a non-receptor protein tyrosine kinase that is mutated and defective in patients with X−linked agammaglobulinemia (XLA) (1–3). BTK is predominantly expressed in hematopoietic cells (4, 5). In B lymphocytes, BTK is primarily required for B cell receptor (BCR) signaling (2, 3, 6). Upon BCR activation, BTK is recruited to the BCR signaling complex, where Btk activity is positively regulated by phosphorylation events. Activated BTK in turn phosphorylates PLCγ2 to induce downstream transcription factors such as NF−κB, NF-AT and ERK1/2 (1–3, 6). In addition to BCR, BTK also participates in the signaling pathways of chemokine receptors in B cells, including CXCR4 and CXCR5 (1, 7). Therefore, BTK plays essential roles in regulating B cell development, survival, proliferation, differentiation, activation and chemotaxis (2, 3, 6). Elevated expression and activity of BTK have been ubiquitously documented in many types of B cell malignancies (1, 8, 9). Aberrant BTK activities play crucial driving roles in the pathogenesis of B cell malignancies, including malignant B cell survival, proliferation and migration, and thus have been recognized as a prime therapeutic target for the treatment of B cell malignancies (1–3, 6).

Successful milestones have been achieved in the pursuit of selective BTK inhibitors. The first-in-class BTK inhibitor ibrutinib and the pioneer second-generation BTK inhibitor acalabrutinib (ACP-196) are two US Food and Drug Administration (FDA)-approved drugs for the treatment of B cell malignances (2, 6, 10). Both ibrutinib and acalabrutinib irreversibly inactivate BTK by covalently binding to Cys481 in the ATP-binding site of BTK (1, 2, 11, 12). The structures of ibrutinib and acalabrutinib as well as their interactions with BTK have been elucidated and depicted in several excellent publications (12–16). Since their approval by FDA, ibrutinib and acalabrutinib have demonstrated clinical efficacy and safety profiles superior to conventional chemoimmunotherapy (CIT) regimens in patients with chronic lymphocytic leukemia (CLL) and relapsed mantle cell lymphoma (MCL), especially in high-risk patients, bringing a major breakthrough in the field (10, 17–20). As a result, ibrutinib and acalabrutinib are currently recommended as the standard of care and preferred choice of treatment in CLL and relapsed MCL, and have also transformed the treatment options for other B cell malignancies (10, 17, 20–23).

Interestingly, mounting preclinical and clinical evidence indicates that both BTK inhibitors are much more versatile than initially envisioned and have direct effects on many cell types beyond B lymphocytes (24). In addition to B cells, many other cell types express BTK under physiological or pathological conditions, including T cells, monocytes, macrophages, granulocytes, myeloid-derived suppressor cells (MDSCs), dendritic cells (DCs), osteoclasts, mast cells, erythrocytes, platelets, epithelial cells, neurons and astrocytes (3, 24–31). Most notably, both ibrutinib and acalabrutinib have complex immunomodulatory effects on various non-B immune cell subsets by inhibiting BTK-dependent signaling pathways of specific immune receptors, including T cell receptor (TCR), Toll-like receptors (TLRs), NLRP3, TREM-1, Dectin-1, CXCR4, CXCR5, RANK, Fc receptors and CLEC-2, among others (2, 3, 6, 24–26, 32, 33). Due to its off-target inhibition of other kinases such as ITK, TEC, the SRC family kinases, EGFR, PDGF-R, VEGF-R2 and CSK, ibrutinib has additional distinct effects on T cells, natural killer (NK) cells, myeloid cells, platelets, epithelial cells, endothelial cells and cardiomyocytes (27–29, 34–40). Such mechanisms of action contribute to the exceptionally high clinical efficacy as well as the unique profiles of adverse effects observed for ibrutinib and acalabrutinib in CLL and MCL patients. Furthermore, these findings have vastly expanded the therapeutic potential of both drugs in human diseases.

Owing to their complex immunomodulatory effects together with the ease of their oral administration, their well-tolerated toxicity profiles and their potential for long-term treatment in patients, ibrutinib and acalabrutinib have become very popular in the management of B cell malignancies (6, 41). Meanwhile, the favorable and versatile features of the two drugs have attracted great interests to explore their repurposing opportunities for the treatment of a wide variety of other human diseases. Over 470 clinical trials are currently registered at ClinicalTrials.gov to test the efficacy of ibrutinib or acalabrutinib not only in almost every type of B cell malignancies, but also in hematological malignancies of myeloid cells and T cells, solid tumors, chronic graft *versus* host disease, autoimmune diseases, allergy and COVID-19 (http:www.clinicaltrials.gov). In this review, we present a brief discussion of the clinical trials and relevant key preclinical evidence of ibrutinib and acalabrutinib as monotherapies or as part of combination therapies for the treatment of human diseases beyond B cell malignancies. We hope such discussion will promote further explorations on the multifaceted therapeutic potential and repurposing opportunities of BTK inhibitors.

## Hematological Malignancies of Myeloid Cells and T Cells

BTK is upregulated and constitutively phosphorylated in the majority of primary AML samples (42–44). In AML cells, BTK phosphorylation can be induced by SCF-CD117 signaling or activating mutations of G-CSFR (T618I or truncated Q741x) or FLT3 (with internal tandem duplication of the juxtamembrane region, FLT3-ITD) to mediate cell survival, proliferation and adhesion, and can also be elicited by SDF1-CXCR4 signaling to mediate cell migration and adhesion to stroma cells (44–47). Inhibition of BTK by ibrutinib or knockdown of BTK by siRNA in primary AML cells and AML cell lines decreases NF-κB survival pathways, SCF-CD117-BTK-MAPK/ATK signaling, G-CSFR mutants-BTK signaling, FLT3-ITD-BTK-NF-κB/MAPK/AKT/STAT5 signaling, and SDF1/CXCR4-BTK-AKT/MAPK signaling, leading to reduced cell survival and proliferation as well as migration (43–48). Together, these data reveal the pathogenic roles of BTK in AML, identifying BTK as a therapeutic target in myeloid cell malignancies.

Based on the above preclinical evidence, two clinical trials are testing the efficacy of ibrutinib for the prevention and treatment of AML and two additional trials are evaluating the effects of ibrutinib in patients with myelodysplastic syndrome (MDS) (**Tables 1** and **2**). Among these, one Phase II trial (NCT03267186) is studying the efficacy of ibrutinib in preventing AML relapse after reduced-intensity conditioning and allogeneic hematopoietic cell transplantation (allo-HCT) for AML. A Phase II/III trial (NCT03642236) is evaluating the potential of ibrutinib in combination with conventional chemotherapy (decitabine, aclacinomycin, cytarabine, G-CSF and sorafenib with or without FLT3 inhibitor) to overcome drug-resistance in relapsed or refractory (R/R) FLT3 mutant AML. For the treatment of MDS, a Phase Ib trial (NCT02553941) is testing the effects of ibrutinib in combination with azacitidine in higher risk MDS patients. Another Phase I trial (NCT03359460) is investigating the efficacy of ibrutinib in combination with lenalidomide in MDS patients that have failed or refused standard therapy.

**Table 1:** Clinical trials of BTK inhibitors in human diseases beyond B cell malignancies that are currently registered at ClinicalTrials.gov.

Diseases	Btk inhibitor	Phase	Trial Identifier
Hematological malignancies of myeloid cells and T cells			
AML			
prevention of relapse after conditioning/Allo-HCT	Ibrutinib	Phase II	NCT03267186
refractory/relapsed FLT3 mutant	Ibrutinib (with conventional chemotherapy,	Phase II/III	NCT03642236
	with or or without FLT3 inhibitor)		
Myelodysplastic syndrome			
failed or refuse standard therapy	Ibrutinib (with Lenalidomide)	Phase I	NCT03359460
higher risk patients	Ibrutinib (with Azacitidine)	Phase Ib	NCT02553941
Mastocytosis	Ibrutinib	Phase II - terminated	NCT02415608
T-cell lymphoma			
relapsed and refractory	Ibrutinib	Phase I	NCT02309580
T-cell prolymphocytic leukemia			
relapsed and refractory	Ibrutinib (with Venetoclax)	Phase II	NCT03873493
Solid tumors			
Breast cancer	Ibrutinib (with MEDI4736)	Phase I/II	NCT02403271
HER2-amplified metastatic	Ibrutinib (with Trastuzumab)	Phase I/II	NCT03379428
Colorectal cancers, advanced, refractory	Ibrutinib (with Pembrolizumab)	Phase I/II	NCT03332498
Gastrointestinal and genitourinary cancer, advanced	Ibrutinib (with everolimus, paclitaxel,	Phase I/II	NCT02599324
	docetaxel, pembrolizumab or cetuximab)		
Glioblastoma	Ibrutinib	Phase I	NCT03535350
recurrent	Acalabrutinib	Phase Ib/II	NCT02586857
Head and neck squamous cell carcinoma	Acalabrutinib (with Pembrolizumab)	Phase II - completed	NCT02454179
recurrent and/or metastatic	Ibrutinib (with Nivolumab or Cetuximab)	Phase II	NCT03646461
Kidney cancer (previously treated, metastatic)	Ibrutinib (with Nivolumab)	Phase I/II	NCT02899078
Lung cancer, non-small cell	Acalabrutinib (with Pembrolizumab)	Phase II - completed	NCT02448303
	Ibrutinib (with MEDI4736)	Phase I/II	NCT02403271
previously treated	Ibrutinib	Phase I/II	NCT02321540
Melanoma	Ibrutinib (with Pembrolizumab)	Phase I	NCT03021460
refractory metastatic	Ibrutinib	Phase II	NCT02581930
Oesophagogastric carcinoma	Ibrutinib	Phase II	NCT02884453
Ovarian cancer	Acalabrutinib	Phase II - completed	NCT02537444
Pancreatic cancer	Ibrutinib (with MEDI4736)	Phase I/II	NCT02403271
advanced or metastatic	Acalabrutinib	Phase II - complete	NCT02362048
adenocarcinoma, metastatic	Ibrutinib (with Nab-paclitaxel	Phase III - completed	NCT02436668
	and Gemcitabine)		
	Ibrutinib (with Gemcitabine	Phase I/II - completed	NCT02562898
	and Nab-Paclitaxel)		
neuroendocrine tumors	Ibrutinib	Phase II - completed	NCT02575300
Prostate cancer (localized)	Ibrutinib (as Neoadjuvant therapy)	Phase I/II	NCT02643667
Urothelial carcinoma	Acalabrutinib (with Pembrolizumab)	Phase II - completed	NCT02351739
Metastatic solid tumors	Ibrutinib (with Nivolumab)	Phase I	NCT03525925
Graft-versus-host disease			
cGVHD	Ibrutinib (as front line)	Phase II	NCT04294641
	Ibrutinib (with Rituximab)	Phase I/II	NCT03689894
	Ibrutinib (with Rituximab)	Phase II	NCT04235036
	Ibrutinib (with Corticosteroids)	Phase III	NCT02959944
steroid dependent/refractory	Ibrutinib	Phase III	NCT03474679
	Ibrutinib	Phase I/II - completed	NCT02195869
steroid refractory	Acalabrutinib	Phase II	NCT04198922
pediatric subjects	Ibrutinib	Phase I/II	NCT03790332
Autoimmune diseases			
Autoimmune hemolytic anemia			
refractory/relapsed	Ibrutinib	Phase II	NCT04398459
steroid refractory, with underlying CLL	Ibrutinib (with Rituximab)	Phase II	NCT03827603
	Acalabrutinib	Phase II	NCT04657094
Rheumatoid arthritis	Acalabrutinib	Phase II - Completed	NCT02387762
Allergic diseases			
Anaphylaxis, food-induced	Ibrutinib	Phase II - completed	NCT03149315
Infectious and inflammatory diseases			
COVID-19	Ibrutinib	Phase II	NCT04375397
COVID-19 patients requiring hospitalization	Ibrutinib	Phase Ib/II	NCT04439006
	Acalabrutinib	Phase III	NCT04647669
	Acalabrutinib	Phase II - completed	NCT04346199
	Acalabrutinib	Phase II - completed	NCT04380688
	Acalabrutinib	Phase I - completed	NCT04564040
COVID-19 in patients with B cell malignancies	Ibrutinib	Phase II	NCT04665115

**Table 2:** Recruiting info and preliminary results of clinical trials with ibruitinib and acalabrutinib in human diseases beyond B cell malignancies.

Clinical trial	Recruitment #	Method of	Sponsor	Location of trial	Trial results	Major toxicities	Ref.
	Actual/Target	recruiment					
Hematological cancers							
NCT03267186	8/50	Site-specific	Andrew Rezvani, Stanford University	United States	Not posted	Not posted	
NCT03642236	122/122	Enrolling by invitation	Nanfang Hospital of Southern Medical University	China	Not posted	Not posted	
NCT03359460	20/20	Site-specific	Brian Jonas	United States	Not posted	Not posted	
NCT02553941	21/24	Not specified	Brian Jonas	United States	Not posted	Not posted	
NCT02415608	4/11 (Terminated due to slow accrual)	Site-specific	Jason Robert Gotlib	United States	ORR to ibrutinib is 0% in 4 patients with advanced systemic mastocytosis.	Fatigue, anemia, gastrointestinal disorders	
NCT02309580	Recruiting/19	Site-specific	Memorial Sloan Kettering Cancer Center	United States	ORR to ibrutinib is 8% in 13 patients with R/R TCL.	Thrombocytopenia, diarrhea, fatigue	(49)
NCT03873493	14/37	Not specified	AbbVie	United States	Ibrutinib plus venetoclax produces clinical responses in two patients with R/R T-PLL, but the results of other patients are not posted.	Not posted	(50)
Solid tumors							
NCT02403271	124/160	Not specified	Pharmacyclics LLC	United States	ORR to ibrutinib in combination with durvalumab is 1.9% in 105 patients with R/R NSCLC, breast or pancreatic cancer.	Fatigue, gastrointestinal disorders, anemia	
NCT03379428	Recruiting/51	Site-specific	US Oncology Research	United States	Not posted	Not posted	
NCT03332498	40/42	Site-specific	H. Lee Moffitt Cancer Center and Research Institute	United States	Disease control rate by ibrutinib plus pembrolizumab is 41.9% in 31 patients with metastatic colorectal cancers, but this trial lacks a control arm with pembrolizumab alone or plus placebo.	Anemia, fatigue, elevated alkaline phosphatase	(51)
NCT02599324	261/189	Not specified	Pharmacyclics LLC	United States	Not posted	Not posted	
NCT03535350	Recruiting/36	Site-specific	Case Comprehensive Cancer Center	United States	Not posted	Not posted	
NCT02586857	24/72	Not specified	Acerta Pharma BV	United States	ORR to 200 mg acalabrutinib is 6.7% in 15 patients and ORR to 400 mg acalabrutinib is 11% in 9 patients with recurrent glioblastoma.	Gastrointestinal disorders, fatique, fall injury	
NCT02454179	78/74	Site-specific	Acerta Pharma BV	United States	ORR is not improved by 100 mg (BID) acalabrutinib plus pembrolizumab (16.7%; 5/30) as compared to pembrolizumab monotherapy (18.9%; 7/37) in patients with advanced HNSCC.	Fatigue, anemia, gastrointestinal disorders, decreased appetite	
NCT03646461	Recruiting/39	Site-specific	University of California, San Diego	United States	Not posted	Not posted	
NCT02899078	31/30	Site-specific	University of California, Davis	United States	Not posted	Not posted	
NCT02448303	74/74	Site-specific	Acerta Pharma BV	United States	ORR is not improved by 100 mg (BID) acalabrutinib plus pembrolizumab (14.3%; 4/28) as compared to pembrolizumab monotherapy (12.9%; 4/31) in patients with advanced NSCLC.	Gastrointestinal disorders, decreased appetite, dyspnoea	
NCT02321540	13/38	Site-specific	M.D. Anderson Cancer Center	United States	Not posted	Not posted	
NCT03021460	23/51; recruiting	Site-specific	Mayo Clinic	United States	Not posted	Not posted	
NCT02581930	18/32	Site-specific	National Cancer Institute (NCI)	United States	ORR to ibrutinib is 0% in 18 patients with refractory metastatic cutaneous melanoma.	Fatigue, anorexia, hyponatremia, gastrointestinal disorders	(52)
NCT02884453	Recruiting/17	Not specified	Royal Marsden NHS Foundation Trust	United Kingdom	Not posted	Not posted	
NCT02537444	78/76	Site-specific	Acerta Pharma BV	United States	ORR to 100 mg (BID) acalabrutinib monotherapy is 2.9% (1/35) and ORR to acalabrutinib plus pembrolizumab is 9.1% (3/33) in patients with refractory ovarian cancer.	Gastrointestinal disorders, headache, fatique, anemia	
NCT02362048	77/76	Site-specific	Acerta Pharma BV	United States	ORR to 100 mg (BID) acalabrutinib monotherapy is 0% (0/37) and ORR to acalabrutinib plus pembrolizumab is 7.5% (3/40) in patients with refractory or metastatic pancreatic cancer.	Gastrointestinal disorders, headache, fatique, anemia, decreased appetite	(53)
NCT02436668	430/326	Global	Pharmacyclics LLC.	United States, European Union, South Korea	As compared to placebo plus gemcitabine/Nab-paclitaxel, ibrutinib plus gemcitabine/Nab-paclitaxel did not improve the progression free survival (PFS) or overall survival (OS) in patients with metastatic pancreatic cancer.	Gastrointestinal disorders, thrombocytopenia	
NCT02562898	18/35	Site-specific	Margaret Tempero	United States	CA19-9 clinical response rate to ibrutinib plus gemcitabine/Nab-paclitaxel is <1% in patients with metastatic pancreatic cancer.	Gastrointestinal disorders, fatique, myalgia, sepsis	(54)
NCT02575300	20/51	Site-specific	H. Lee Moffitt Cancer Center	United States	ORR to ibrutinib is 0% in 20 patients with advanced carcinoid and pancreatic neuroendocrine tumors	Gastrointestinal disorders, fatique, arthralgia	
NCT02643667	Recruiting/36	Site-specific	Washington University School of Medicine	United States	Not posted	Not posted	
NCT02351739	78/74	Not specified	Acerta Pharma BV	United States	ORR is not improved by 100 mg (BID) acalabrutinib plus pembrolizumab (23.5%; 8/34) as compared to pembrolizumab monotherapy (29%; 9/31) in patients with platinum resistant urothelial bladder cancer.	Fatique, gastrointestinal disorders, increased alanine aminotransferase	(55)
NCT03525925	15/15	Site-specific	Ohio State University Cancer Center	United States	Not posted	Not posted	
Graft-versus-host disease							
NCT04294641	Recruiting/40	Site-specific	National Cancer Institute (NCI)	United States	Not posted	Not posted	
NCT03689894	Recruiting/15	Site-specific	Dartmouth-Hitchcock Medical Center	United States	Not posted	Not posted	
NCT04235036	Recruiting/35	Site-specific	Northside Hospital, Inc.	United States	Not posted	Not posted	
NCT02959944	193/186	Global	Pharmacyclics LLC.	14 different countries	ORR is minimally improved by ibrutinib plus prednisone (41.1%; 39/95) as compared to placebo plus prednisone (36.7%; 36/98) in patients with new onset cGVHD.	Peripheral edema, insomnia, thrombocytopenia	
NCT03474679	19/17	Not specified	Janssen Pharmaceutical K.K	Japan	Not posted	Not posted	
NCT02195869	45/39	Not specified	Pharmacyclics LLC.	United States	ORR to ibrutinib is 69% in 42 patients with cGVHD who were steroid-dependent or -refractory.	Fatigue, gastrointestinal disorders, muscle spasms, bruising, pneumonia	(56, 57)
NCT04198922	Recruiting/50	Not specified	Fred Hutchinson Cancer Research Center	United States	Not posted	Not posted	
NCT03790332	58/44	Global	Pharmacyclics LLC.	14 different countries	Not posted	Not posted	(58)
Autoimmune diseases							
NCT04398459	Recruiting/18	Site-specific	Institute of Hematology & Blood Diseases Hospital	China	Not posted	Not posted	
NCT03827603	50/50	Site-specific	Eugene Nikitin	Russian Federation	Not posted	Not posted	
NCT04657094	Recruiting/22	Site-specific	City of Hope Medical Center	United States	Not posted	Not posted	
NCT02387762	31/70	Not specifed	Acerta Pharma BV	United States	Disease activity score 28-CRP (DAS28-CRP) at Week 4 is not improved by 15 mg (QD) acalabrutinib plus methotrexate (5.40; n=15) as compared to placebo plus methotrexate (5.05; n=15) in patients with RA.	Anemia	
Allergic diseases							
NCT03149315	6/6	Site-specific	Ann & Robert H Lurie Children's Hospital of Chicago	United States	Short-term ibrutinib therapy suppreses skin test responses and eliminates IgE-mediated basophil activation in 6 patients with an allergy to peanut or tree nuts (ORR: 100%).	No common side effects observed	(59)
COVID-19							
NCT04375397	46/46	Not specified	Abbvie	United States	Not posted	Not posted	
NCT04439006	Recruiting/78	Site-specific	Jennifer Woyach, Ohio State University Cancer Center	United States	Not posted	Not posted	
NCT04647669	Not yet recruiting/100	Site-specific	The University of The West Indies	Jamaica	Not posted	Not posted	
NCT04346199	177/428	Global	AstraZeneca	Worldwide	Percentage of participants that remained alive and free of respiratory failure is not improved by acalabrutinib plus best supportive care (BSC) (n=89) as compared to BSC alone (n=88) in patients hospitalized with COVID-19.	Headache	
NCT04380688	62/60	Not specified	AstraZeneca	United States	Percentage of participants that remained alive and free of respiratory failure is not improved by acalabrutinib plus BSC (n=31) as compared to BSC alone (n=31) in patients hospitalized with COVID-19.	Headache	
NCT04564040	20/40	Site-specific	AstraZeneca	Germany	Not posted	Not posted	
NCT04665115	Not yet recruiting/134	Site-specific	Academic and Community Cancer Research United	United States	Not posted	Not posted	

However, a recently completed Phase IIa clinical trial (NCT02351037) showed limited efficacy of ibrutinib alone or in combination with cytarabine or azacitidine in patients with AML (60). Another completed Phase II clinical trial (registered in the Netherlands NL5751 [NTR6017] and EudraCT number 2015-002855-85) reported that addition of ibrutinib to decitabine does not improve the therapeutic efficacy of decitabine in unfit patients with AML and higher risk MDS (61). Although the efficacy of ibrutinib in AML and MDS awaits clarification with additional clinical data from ongoing trials, it is possible that ibrutinib may be effective only in combination with appropriate drugs or only for patients with specific oncogenic mutations or genetic alterations. In this regard, Li et al. found that ibrutinib acts synergistically with the cytotoxic alkaloid homoharringtonine to inhibit cell proliferation and induce apoptosis in AML cells with FLT3-ITD mutations (62). Another study by Eide et al. recently reported that the primary AML samples with 11q23 MLL rearrangements are highly sensitive to ibrutinib in combination with the BCL-2 inhibitor venetoclax (63). Furthermore, both ibrutinib and acalabrutinib exhibit synergistic effects with daunorubicin on inducing cytotoxicity in AKR1C3-expressing AML cells *via* efficiently preventing daunorubicin inactivation mediated by AKR1C3, an enzyme associated with the emergence of multidrug resistance (MDR) (64). Therefore, clinical data from ongoing trials and better understanding of the mechanisms of action for ibrutinib and acalabrutinib will guide the design and improvement of future trials, including optimization of the combination regimens and appropriate patient stratification according to relevant genetic alterations and contexts.

In addition to AML and MDS, aberrant activation of BTK has been detected in patients of chronic neutrophilic leukemia (CNL) with G-CSFR mutations and these cells show high sensitivity to ibrutinib treatment (45). Ibrutinib also suppresses BTK phosphorylation, induces apoptosis and decreases proliferation in canine neoplastic mast cells (65). Although a Phase II clinical trial (NCT02415608) of ibrutinib in systemic mastocytosis was recently terminated due to slow accrual, these preclinical findings suggest a potential of ibrutinib and other BTK inhibitors as new therapeutic agents for CNL and mast cell neoplasms.

Because of its off-target inhibition on ITK, ibrutinib is being tested in T cell malignancies (**Tables 1** and **2**). One Phase I clinical trial (NCT02309580) is investigating the efficacy of ibrutinib in adult patients with R/R T-cell lymphoma (TCL), including peripheral TCL (PTCL) and cutaneous TCL (CTCL). Another Phase II clinical trial (NCT03873493) is evaluating the efficacy of ibrutinib in combination with venetoclax in adult patients with R/R T-cell prolymphocytic leukemia (T-PLL). Mechanistically, ITK is required for TCR signaling and CXCR4 signaling, and thus critical for malignant T cell proliferation, differentiation and migration, while preventing anti-tumor immune responses by inhibiting T_H_1 CD4 T cell differentiation (49, 50, 66). Treatment with ibrutinib or knockdown of ITK by siRNA results in reduced ITK phosphorylation and decreased activation of downstream MEK1/2 and AKT, leading to compromised survival and cytokine production as well as migration of PTCL cells (66). A large proportion of PTCL cases are derived from follicular helper T cells (Tfh), which express high levels of ITK proteins (66). In a mouse model of Tfh-derived lymphoma, ibrutinib effectively induces lymphoma regression (67). However, the preliminary results of the Phase I trial NCT02309580 suggest that ibrutinib has limited clinical benefits in 13 patients with R/R TCL (49) (**Table 2**). Interestingly, although ibrutinib alone has very modest effects on T-PLL cells, the combination of ibrutinib and the BCL-2 inhibitor venetoclax exhibits strong synergism at inducing apoptosis in primary T-PLL cells (50). This synergism is because venetoclax monotherapy leads to ITK activation and ibrutinib-mediated ITK inhibition enhances the dependence of T-PLL cells on BCL-2 for survival (50). An anecdotal case of combinatorial treatment of one T-PLL patient was recently published by Oberbeck et al., which reported disease stabilization in the patient after short-term treatment with ibrutinib plus venetoclax but progression after cessation of treatment (68). In line with this, preliminary clinical data of the Phase II trial NCT03873493 demonstrated that ibrutinib in combination with venetoclax produces clinical responses in two patients with R/R T-PLL (50). Thus, it is promising that more clinical data from ongoing trials will advance the development and addition of ibrutinib into the treatment regimens for T cell malignancies.

## Solid Tumors

Currently, 24 clinical trials are registered at ClinicalTrials.gov to test the efficacy of ibrutinib (18 trials) and acalabrutinib (6 trials), alone or in combination therapy, in patients with a variety of solid tumors, including breast, prostate, lung, kidney, head and neck, pancreatic, colorectal, oesophagogastric, urothelial, ovarian, gastrointestinal and genitourinary cancers, glioblastoma, melanoma and metastatic solid tumors (**Tables 1** and **2**). These clinical trials are elicited by strong preclinical evidence indicating that ibrutinib and acalabrutinib will potentially have broad applications in the treatment of various solid tumors because of their multi-layered mechanisms of action (24, 28, 29, 53, 69–79). These include both direct tumoricidal activities on cancer cells and indirect immunomodulatory effects on different immune cell subsets as well as other relevant cell types in the tumor microenvironment (TME) *via* on-target inhibition of BTK (for both drugs) or off-target inhibition of ITK or EGFR (for ibrutinib) (2, 24, 28, 29).

Ibrutinib and acalabrutinib exhibit direct tumoricidal activities on certain types of solid tumor cells, including neuroblastoma, glioblastoma, breast cancer, prostate cancer, bladder cancer and advanced oral squamous cell carcinoma (OSCC) (30, 31, 78, 80–82). Accumulating studies report that BTK is highly expressed in certain solid tumor cells and increased BTK levels are associated with a poor prognosis in patients (30, 31, 78, 80–82). Gene silencing of BTK or inhibition of BTK with ibrutinib or acalabrutinib attenuates the proliferation, migration, invasion and stemness of these cancer cells both *in vitro* and *in vivo* (30, 31, 78, 80–82). Mechanistically, ibrutinib or acalabrutinib potently inhibits BTK phosphorylation and its downstream oncogenic pathways such as the PI3K-mTOR and MEK/MAPK pathways, resulting in impaired tumorigenicity of these tumor cells (30, 31, 78, 80–82). However, it should be noted that there are controversial reports regarding the expression of BTK in tumor cells derived from non-hematopoietic lineages. For example, Li et al. reported that the expression of BTK is detectable in human neuroblastoma cell lines IMR32, LAN2, NBL-S and SHSY5Y (83), and Pikatan et al. also detected the expression of BTK in human neuroblastoma cell lines SK-N-BE2 and SH-5YSY by Western blot analysis (30). In contrast, Ishfaq et al. reported that the expression of BTK is not detectable in human neuroblastoma cell lines SK-N-BE2, IMR32, SH-SY-5Y and SKNSH or a murine neuroblastoma cell line NB9464 by Western blot analysis (84). In addition to the contradictory observations in BTK expression, these three groups also reported contradictory effects of ibrutinib on neuroblastoma cell lines. Ishfaq et al. did not observe any effect of ibrutinib on the proliferation of murine and human neuroblastoma cell lines (84). In sharp contrast, Li et al. and Pikatan et al. reported that silencing of BTK by siRNAs or inhibition of BTK by ibrutinib or acalabrutinib significantly reduces the proliferation and viability of human neuroblastoma cell lines (30, 83). It remains unclear what factors may cause such contradictory results in the same cell lines. In this context, the expression of BTK in solid tumors derived from non-hematopoietic lineage cells requires more stringent scrutiny in future studies, especially given the frequent presence of BTK-expressing infiltrating immune cells in the TME of primary tumor specimens.

Indeed, BTK is critical for the function of multiple cell types representing important components of the TME, including macrophages, MDSCs, DCs, neutrophils, B cells and endothelial cells (2, 24, 28, 29). In particular, BTK is overexpressed in TAMs and MDSCs, which regulate tumor progression, immunosuppression and angiogenesis (71, 84, 85). Ibrutinib treatment, *Btk* deficiency or siRNA-mediated knockdown of BTK shifts macrophage polarization from tumor-promoting M2-like macrophages toward inflammatory M1-like and diminishes PD-1 and SIRPα expression on macrophages (24, 77, 86, 87). Ibrutinib treatment also inhibits MDSC generation, induces MDSC differentiation to mature DCs, and impairs IDO expression and NO production in MDSCs, resulting in reduced MDSC-mediated immunosuppression and increased CD8 T cell proliferation, infiltration and effector function in the TME (71, 80, 84, 88). These observations are consistent with the previous evidence that *Btk* ^-/-^ DCs exhibit a more mature phenotype, expressing higher levels of MHC class II and co-stimulatory molecules, than wild type DCs (89, 90). Interestingly, emerging evidence reveals complex roles of different B cell subsets in the progression of solid tumors (77, 91, 92). BTK regulates the crosstalk between B cells and FcRγ+ TAMs in the TME and thus TAM-mediated immunosuppression of T cells in pancreatic adenocarcinomas, which can be reverted by treatment with ibrutinib or siRNA-mediated knockdown of BTK (77). Ibrutinib also inhibits the production of immunosuppressive adenosine by regulatory B cells (Breg) and increases the infiltration of effector B cells into the TME (74). Taken together, ibrutinib-mediated inhibition of BTK in TAMs, MDSCs, DCs and B cells indirectly promote anti-tumor immunity by augmenting T_H_1 CD4 T cell response and increasing the infiltration of CD8 cytotoxic T cells and effector B cells into the TME (24, 69, 74, 77, 80, 84, 93).

An additional indirect anti-tumor mechanism *via* on-target inhibition of BTK by ibrutinib or acalabrutinib is realized through the regulation of cytokines, chemokines and growth factors. *Btk* deficiency or inhibition of BTK with ibrutinib or acalabrutinib impairs TLR signaling and inflammasome activation in TAMs, MDSCs, DCs and B cells, thereby efficiently suppressing the production of specific cytokines, chemokines and growth factors such as IL-1β, IL-6, CXCL12, CXCL13, CCL19 and VEGF (2, 24, 28, 29, 69, 94, 95). Reduced levels of these molecules significantly compromise the adhesion, migration and invasion of tumor cells, and also impair the ability of endothelial cells to undergo angiogenic tube formation (2, 28, 29, 69, 95). Thus, ibrutinib and acalabrutinib can be of clinical use in abrogating inflammation-associated cancer progression and angiogenesis in the TME (2, 28, 29, 69, 95).

It has been well-documented that off-target inhibition of ITK allows ibrutinib to modulate CD4 T cell differentiation and CD8 T cell effector function (24, 28, 29, 34, 96). ITK is required for CD4 T_H_2 differentiation and negatively regulates the expression of the transcription factor Eomes in CD8 T cells (34, 96). Eomes inhibits the expression of checkpoint receptors (PD-1, TIM-3, and LAG-3) and induces the expression of effector cytokines (IFNγ and TNFα) in CD8 T cells (96). Therefore, ibrutinib-mediated inhibition of ITK can robustly enhance anti-tumor immune responses by favoring T_H_1 differentiation and promoting the effector functions of CD8 cytotoxic T cells (96). On the other hand, off-target inhibition of EGFR allows ibrutinib to directly suppress the proliferation, growth and stemness of certain types of solid tumors that are dependent on EGFR oncogenic pathways, including pancreatic cancer, hepatocellular carcinoma (HCC) and esophageal squamous cell carcinoma (ESCC) (97–99). Ibrutinib represses the phosphorylation of EGFR and thus inhibits the downstream activation of AKT and ERK signaling in these cancer cells, leading to reduced tumorigenicity both *in vitro* and *in vivo* (97–99). In addition, off-target inhibition of EGFR by ibrutinib also abrogates the pro-tumoral function of glioma-derived pericytes with EGFR mutations (100). Thus, through its off-target inhibition of EGFR in cancer cells or pericytes and ITK in T cells, ibrutinib can directly suppress the tumorigenicity and growth of EGFR-dependent solid tumors and dampen the pro-tumoral function of pericytes with EGFR mutations, while indirectly promoting anti-tumor T_H_1 and CD8 T cell responses to attenuate tumor progression.

Both their direct cytotoxic effects on tumor cells and their indirect immunomodulatory effects on various immune cell subsets in the TME provide strong rationales for a potentially broad use of ibrutinib and acalabrutinib in solid tumors (2, 28, 29, 69). However, preliminary clinical data of several trials show no or limited clinical benefit of ibrutinib and acalabrutinib at improving the objective response rates (ORRs), progression-free survival (PFS) or overall survival (OS) in patients with several solid tumors (**Table 2**). These include ibrutinib plus durvalumab in R/R non-small cell lung cancer (NSCLC), breast cancer and pancreatic cancer (NCT02403271); acalabrutinib in recurrent glioblastoma (NCT02586857); ibrutinib in refractory metastatic cutaneous melanoma (NCT02581930) (52); acalabrutinib in combination with pembrolizumab in advanced head and neck squamous cell carcinoma (HNSCC) (NCT02454179), non-small cell lung cancer (NCT02448303) and platinum-refractory metastatic urothelial carcinoma (NCT02351739) (55); acalabrutinib alone or in combination with pembrolizumab in refractory ovarian cancer (NCT02537444) and metastatic or locally advanced unresectable pancreatic ductal adenocarcinoma (NCT02362048) (53); ibrutinib in combination with Nab-paclitaxel and gemcitabine in metastatic pancreatic cancer (NCT02436668) and (NCT02562898) (54); and ibrutinib alone in advanced carcinoid and pancreatic neuroendocrine tumors (**Table 2**) (www.clinicaltrials.com). Nonetheless, peripheral reductions in MDSCs and increases in proliferating CD8 T cell subsets are observed in some of these cases (53, 55). It has been noticed that lack of efficacy is at least partially due to the rapid clearance *in vivo* and low accumulation of ibrutinib and acalabrutinib in solid tumors (12, 85). To circumvent this hurdle, Qiu et al. has recently developed novel nanocomplexes coated with a sialic acid (SA)-stearic acid conjugate-decorated surface to encapsulate ibrutinib, which demonstrates prolonged blood circulation and efficient delivery of ibrutinib to the tumor, consequently enhancing anti-tumor immunity, reducing angiogenesis and suppressing tumor growth (85). Therefore, the challenging tasks in future clinical trials are to develop more efficient delivery strategies, implement better patient stratification according to their genetic contexts and tumor stage, and optimize the regimens as well as the sequence of combination therapies to improve the efficacy of ibrutinib and acalabrutinib in the treatment of solid tumors.

## Chronic Graft *Versus* Host Disease (cGVHD)

Based on the favorable results of a completed clinical trial (NCT02195869), ibrutinib has been approved by the FDA in 2017 for the treatment of chronic graft *versus* host disease (cGVHD) in adult patients after failure of one or more first-line therapy (56, 57, 101–103). cGVHD is one of the major complications in patients undergoing allogeneic hematopoietic stem cell transplantation (allo-HSCT) and represents a significant cause of morbidity and mortality after allo-HSCT (104, 105). cGVHD often requires enduring immunosuppressive treatment and corticosteroids remain the cornerstone therapy (103, 104). For steroid-refractory cGVHD, ibrutinib has become one of the preferred options (103, 105, 106). Mechanistically, the key kinases BTK and ITK are critical to cGVHD development, as animals lacking BTK or ITK do not develop alloantibody-driven cGVHD and T cell-mediated sclerodermatous cGVHD (107). Ibrutinib inhibits BTK in B cells and ITK in T cells that mediate the pathogenesis of cGVHD, leading to reduced B cell and T cell proliferation and co-stimulatory molecule activation, decreased germinal center (GC) reactions and tissue immunoglobulin (Ig) deposition, T_H_2 cell depletion, reduced cytokine production and lymphocyte infiltration, ameliorated skin lesions and multiorgan inflammation and fibrosis, which all contribute to improved patient survival (103–105, 107–109).

Currently, there are 7 additional clinical trials of ibrutinib (6 trials) and acalabrutinib (1 trial) in cGVHD registered at ClinicalTrials.gov (**Tables 1** and **2**). One Phase II clinical trial (NCT04198922) is testing the efficacy of acalabrutinib for the treatment of cGVHD caused by stem cell transplants in steroid-refractory adult patients. A phase III clinical trial (NCT03474679) is comparing the effects of ibrutinib between steroid-dependent and steroid-refractory cGVHD patients. Four ongoing clinical trials of ibrutinib are testing the efficacy of ibrutinib as front-line therapy for cGVHD in patients that receive a stem cell or bone marrow transplant, either alone in monotherapy (NCT04294641) or in combination therapy with corticosteroids (NCT02959944) or rituximab (NCT04235036: in newly diagnosed cGVHD; NCT03689894: in steroid-dependent and steroid-refractory cGVHD). Moreover, initial clinical data of ibrutinib in pediatric patients with cGVHD are promising and one Phase I/II clinical trial (NCT03790332) is determining the optimal dosing and further evaluating the efficacy and safety of ibrutinib in pediatric cGVHD patients after failure of 1 or more lines of systemic therapy (58).

Compared to the standard immunosuppressive therapy of cGVHD, corticosteroids (103, 104), ibrutinib has different mechanisms of action and toxicity profiles. Corticosteroids are synthetic analogues of the natural steroid hormones produced by the adrenal cortex. Commonly used glucocorticoids (such as prednisone, prednisolone and dexamethasone) are predominantly involved in carbohydrate, lipid and protein metabolism, and have immunosuppressive, anti-inflammatory, anti-proliferative, and vasoconstrictive effects (110). Most of the immunosuppressive and anti-inflammatory actions of glucocorticoids are mediated by their interactions with the glucocorticoid receptors, which subsequently alter gene transcription in both inflammatory leukocytes and structural cells (such as epithelial and endothelial cells) (110). The well-known adverse effects of corticosteroids include osteoporosis, fractures, adrenal suppression, hyperglycemia, diabetes, cushingoid appearance, weight gain, cataracts, glaucoma, dyslipidemia, psychiatric and cognitive disturbances (110), which are not commonly observed in ibrutinib-treated patients (38) (**Table 2**). Interestingly, the results of NCT02195869 demonstrated that ibrutinib is effective for both corticosteroid-dependent and corticosteroid-refractory cGVHD patients (56, 57). Because of their complementary mechanisms of action, ibrutinib and corticosteroids were hypothesized to have combinatory effects on cGVHD, which would improve clinical benefits in cGVHD patients. However, the trial results of NCT02959944 showed that the ORR is minimally improved by ibrutinib plus prednisone (41.1%) as compared to placebo plus prednisone (36.7%) in patients with new onset cGVHD (**Table 2**). These data together with the results of other ongoing trials will provide new insights to better design future trials to improve the efficacy and safety of ibrutinib as monotherapy or in combination therapy with corticosteroids for the treatment of cGVHD.

In addition to cGVHD, preclinical evidence demonstrates the efficacy of ibrutinib at alleviating the disease manifestations in mouse models of acute GVHD (aGVHD) and allo-skin transplantation, suggesting potential expansion of its use to the treatment of aGVHD and allo-skin graft rejection (108, 111).

## Autoimmune Diseases

Increased BTK protein expression in patients with systemic autoimmune diseases appears to be correlated with autoantibody production (3, 112, 113). Mechanistically, BTK is required for the proliferation and activation of autoreactive B cells (3, 112, 113). Although BTK expression was not initially detected in T cells (114) and XLA patients with *BTK* mutations do not exhibit apparent T cell defects (115–117), Xia et al. recently reported that BTK is indeed expressed in T cells and further upregulated in effector and memory T cells (25). *Btk* ^-/-^ T cells exhibit defective proliferation and reduced expression of the activation marker CD69 as well as production of cytokines following CD3 and CD28 stimulation (25). Inhibition of BTK by acalabrutinib suppresses CD3 plus CD28-induced proliferation of WT but not *Btk* ^-/-^ T cells *in vitro* (25). Notably, *Btk* ^-/-^ donor T cells fail to mount graft-*versus*-host responses and cannot cause bone marrow destruction or blood pancytopenia in a mouse model of autoimmune aplastic anemia (25). This recent finding, which needs to be further confirmed, suggests that BTK may also be implicated in the proliferation and activation of autoreactive T cells. Furthermore, BTK may promote autoimmunity as an important driver of myeloid cell inflammation, osteoclast differentiation and altered B cell-T cell interactions (3, 112, 113). Together, these findings reveal multiple pathogenic roles of BTK in autoimmune diseases.

Based on the above evidence of the pathogenic roles for BTK in autoimmunity, 4 clinical trials are testing the efficacy of ibrutinib (2 trials) and acalabrutinib (2 trials) in autoimmune diseases, including autoimmune hemolytic anemia (AIHA) and rheumatoid arthritis (RA) (**Tables 1** and **2**). One Phase II clinical trial (NCT04398459) is evaluating the safety and efficacy of ibrutinib in R/R AIHA patients that are previously treated with glucocorticoids and rituximab. Two Phase II trials are assessing the efficacy of ibrutinib (NCT03827603) and acalabrutinib (NCT04657094) in R/R AIHA patients with underlying CLL. A recently completed clinical trial (NCT02387762) compared the effects of acalabrutinib *versus* placebo in combination with methotrexate in RA patients, but the initial results of primary outcome do not show beneficial effects of acalabrutinib (**Table 2**). Interestingly, the preliminary results of a Phase IIb clinical trial (NCT03233230) on another BTK inhibitor evobrutinib (also known as M2951 or MSC2364447C) demonstrate that it is effective in RA patients with an inadequate response to methotrexate (www.clinicaltrials.gov). Additional clinical data from these trials will inform the design of future trials to improve the efficacy of BTK inhibitors, alone or in combination with other drugs, for the treatment of AIHA and RA.

Several clinical trials are studying the effects of other BTK inhibitors such as evobrutinib, tirabrutinib (also called ONO/GS-4059) and AC0058TA for the treatment of systemic lupus erythematosus (SLE) and relapsing multiple sclerosis (MS) (www.clinicaltrials.gov) (3, 112, 118–120). In addition, preclinical evidence has shown that ibrutinib effectively ameliorates disease symptoms in patient samples or animal models of SLE, MS, systemic sclerosis (SSc), neuropathy with anti-myelin-associated glycoprotein (MAG), type 2 diabetes (T2D) and obesity (118, 119, 121–125). Such ongoing clinical studies and preclinical evidence demonstrate the immunosuppressive effects of BTK inhibitors in autoimmune diseases and warrant further considerations of new clinical trials on BTK inhibitors as therapeutic agents for these autoimmune diseases.

Commonly used and recently developed drugs for autoimmune diseases include glucocorticoids, non-steroidal anti-inflammatory drugs (NSAIDs; such as aspirin, acetaminophen and selective COX-2 inhibitors), conventional disease-modifying anti-rheumatic drugs (cDMARDs; such as methotrexate and leflunomide), mTOR inhibitors (such as sirolimus and everolimus), JAK inhibitors (such as tofacitinib and baricitinib), biologics (such as etanercept, anakinra and abatacept) and monoclonal antibodies (such as rituximab, infliximab and belimumab) (126, 127). All these drugs have different mechanisms of action and toxicity profiles (126, 127). Traditional NSAIDs mainly inhibits the activity of COX-1 and COX-2, two enzymes responsible for the production of prostaglandins, which mediate inflammation and pain (126). The representative cDMARD methotrexate is an antagonist of folic acid and able to competitively inhibit the activity of folate-dependent enzymes required for DNA and RNA synthesis (126). mTOR inhibitors target essential metabolic pathways in autoimmune lymphocytes such as CD4 T cells (127). JAK inhibitors suppresses the JAK-STAT-dependent signaling pathways of many inflammatory cytokines (127). Biologics typically block specific inflammatory cytokines or immune receptors, including TNFα (such as etanercept), IL-1 (such as anakinra) and CD80/CD86 (such as abatacept), etc. (126, 127). Monoclonal antibodies act to deplete autoimmune B cells (such as rituximab) or block specific inflammatory cytokines (such as infliximab for TNFα, secukinumab for IL-17A and belimumab for BAFF) or immune receptors (such as natalizumab and vedolizumab for integrins) (126, 127). Compared to the above drugs, BTK inhibitors have distinct mechanisms of action. In addition, oral administration of BTK inhibitors is more convenient than subcutaneous injection or *i.v.* infusion required for biologics or monoclonal antibodies. Therefore, if their efficacy in autoimmune diseases is proven by ongoing trials, BTK inhibitors will significantly enrich the treatment options for patients with autoimmune diseases, especially given the possibility of various combination therapies.

## Allergy

One recently completed Phase II clinical trial (NCT03149315) has tested the efficacy of ibrutinib on inhibiting food-induced anaphylaxis in adult patients. The initial results of this trial showed that short-term ibrutinib therapy (as few as 2 doses) suppresses skin test responses and eliminates IgE-mediated basophil activation in adults with peanut or tree nut allergy (59, 128). Mechanistically, BTK is essential for FcϵRI signaling and allergic responses in human mast cells and basophils (59, 128–131). Bone marrow-derived mast cells of *Btk* ^-/-^ mice exhibit impaired FcϵRI-mediated production of eicosanoid, leukotriene C4 (LTC4) and reactive oxygen species (ROS) *in vitro* (132). Both ibrutinib and acalabrutinib can prevent IgE-mediated degranulation, histamine release, cytokine production and upregulation of activation markers (such as CD63, CD164, CD203c or LAMP1) in primary human mast cells and basophils, and thus block allergen-induced contraction of isolated human bronchi *in vitro* (59, 129–131). Preclinical evidence demonstrates that two oral doses of acalabrutinib potently inhibits anaphylaxis in a humanized mouse model of systemic anaphylaxis by suppressing IgE-evoked activation of mast cells and basophils *in vivo*, and also significantly protects mice against death during severe anaphylaxis (129). In addition, ibrutinib attenuates both T_H_2/T_H_17 and neutrophilic/eosinophilic airway inflammation in a mouse model of CAE-induced mixed granulocytic asthma by inhibiting BTK phosphorylation in neutrophils and ITK activation in CD4 T cells (133). Thus, these clinical data and preclinical evidence support additional clinical trials of ibrutinib and acalabrutinib in allergic diseases.

Compared to other drugs commonly used for allergy, ibrutinib and acalabrutinib offer several unique benefits, including the ability to effectively inhibit IgE-FcϵRI-mediated activation of both mast cells and basophils, rapid onset of action and transient efficacy (59, 128). Other commonly used allergy drugs do not have the capability to effectively reduce both mast cell and basophil activation (59, 128). For example, antihistamines target only one mediator, histamine, of allergy and cannot prevent anaphylaxis, which could be triggered by other mediators (such as prostaglandins, leukotrienes and cytokines) released by activated mast cells and basophils (59, 128). Anti-IgE immunotherapy such as omalizumab has shown efficacy in reducing circulating IgE to improve urticaria and asthma, but they cannot completely suppress IgE-dependent mast cell and basophil activation (128). Although omalizumab has been shown to increase the threshold dose of allergen consumption in food-allergic subjects, it takes weeks to months for omalizumab to obtain efficacy. In contrast, two doses of ibrutinib effectively reduce or eliminate skin prick test responses to foods and aeroallergens in allergic subjects (59, 128). Within 1 week after cessation of ibrutinib, these responses are returned to baseline. This transient efficacy suggests that short courses or episodic treatment of ibrutinib could be used to prevent anaphylaxis to foods or drugs (59, 128). However, BTK inhibitors would not likely have efficacy on IgE-independent allergic diseases, as BTK is not known to be involved in IgE-independent pathways of mast cell or basophil activation (128). Because of their distinct mechanisms of action, both ibrutinib and acalabrutinib have the potential to improve the efficacy and reduce adverse events of other commonly used allergy drugs in combination therapies for patients with IgE-dependent allergic diseases.

In addition to ibrutinib and acalabrutinib, several other BTK inhibitors are currently in clinical trials as potential drugs for chronic spontaneous urticaria (CSU) (128). Phase IIa data from a clinical trial (NCT03137069) recently showed impressive efficacy for fenebrutinib (GDC-0853), a highly selective non-covalent BTK inhibitor, at improving clinical scores in patients with antihistamine-refractory CSU, especially in a subgroup of patients with auto-antibodies to FcϵRI, who are refractory to current CSU therapies including omalizumab (128). Another covalent BTK inhibitor, remibrutinib (LOU064), is currently being tested in a Phase IIb clinical trial in patients with antihistamine-resistant CSU (NCT03926611) (128). Collectively, the above preclinical evidence and clinical studies have paved the way for additional clinical trials of ibrutinib and acalabrutinib as well as other BTK inhibitors for the treatment of IgE-dependent anaphylaxis, food allergy, drug allergy, asthma, CSU and other difficult-to-treat allergic diseases.

## COVID-19, Sepsis and Other Infectious and Inflammatory Diseases

Notably, 7 clinical trials are registered to examine the efficacy of acalabrutinib (4 trials) and ibrutinib (3 trials) on COVID-19 (**Tables 1** and **2**), the current pandemic caused by the SARS-CoV-2 virus that has posed a global health threat concerning high mortality rate, economic meltdown and daily life distress (134, 135). Respiratory failure due to acute respiratory distress syndrome (ARDS) is the leading cause of mortality (136, 137). The main mechanism of ARDS is uncontrolled systemic inflammatory response, termed cytokine storm (136, 137). Given the suppressive effects of BTK inhibitors or *Btk* deficiency on cytokine production and inflammatory responses (24, 94), the clinical trials on COVID-19 are evaluating if acalabrutinib or ibrutinib can lessen the inflammatory responses in the lungs and reduce respiratory failure in patients, while preserving overall immune function (138–143). Emerging clinical evidence shows that both acalabrutinib and ibrutinib have protective effects against pulmonary injury, decreasing the duration of mechanical ventilation and mortality rate for hospitalized patients with severe COVID-19 (138–141). The protective effects of BTK inhibitors have also been observed in CLL patients with COVID-19, as the hospitalization rate and duration for severe COVID-19 is lower and shorter for CLL patients on ibrutinib *versus* those on other regimens or off treatment (142, 144–146).

**Figure 1:**
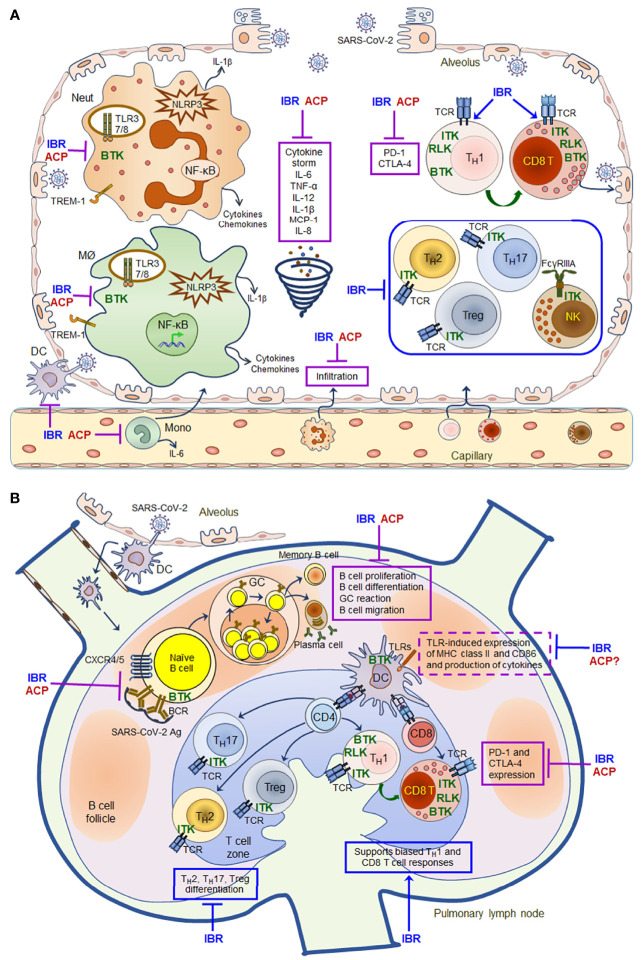
Schematic mechanisms of action for ibrutinib and acalabrutinib in the lung of patients with severe COVID-19. A model of mechanisms of action for ibrutinib (IBR) and acalabrutinib (ACP) is proposed based on the evidence that both drugs have protective effects on ARDS in patients with severe COVID-19 and that both drugs have multifaceted immunomodulatory effects on different immune cell subsets. **(A)** Inhibitory effects of ibrutinib and acalabrutinib on cytokine storm in the alveolus. **(B)** Immunomodulatory effects of ibrutinib and acalabrutinib on B cell and T cell responses in pulmonary lymph nodes. Both drugs can inhibit monocyte, macrophage and neutrophil activation induced by TLRs, NLRP3, TREM-1 and Dectin-1 *via* BTK-dependent mechanisms, thereby decreasing the production of inflammatory cytokines and chemokines such as IL-6, TNFα, IL-12, IL-1β, MCP-1 and IL-8 to ameliorate cytokine storm. Both drugs also inhibit the expression of PD-1 and CTLA-4 on activated T cells *via* BTK-dependent mechanisms, thus enhancing T cell anti-viral immunity. In addition, ITK-dependent effects of ibrutinib on T cell differentiation, effector function and survival may also contribute to its modulatory effects on immunopathology and lymphopenia in COVID-19 therapy, skewing T cells to a T_H_1- and CD8 cytotoxic T cell-dominant populations by inhibiting T_H_2/T_H_17/Treg differentiation, and thus promoting T_H_1 immunity and CD8 cytotoxic effector functions. However, both drugs can suppress the proliferation, differentiation, GC reaction and migration of SARS-CoV-2-specific B cells by inhibiting BCR and CXCR4/CXCR5 signaling *via* BTK-dependent mechanisms, thereby inhibiting the production of antibodies and memory B cell responses. All the shared effects of both drugs are depicted in solid purple boxes, while the distinct ITK-dependent effects of ibrutinib are depicted in solid blue boxes in the figure. Ibrutinib (and likely acalabrutinib) can also affect TLR-induced DC activation and expression of MHC class II and CD86 *via* BTK-dependent mechanisms (depicted in dashed purple boxes in the figure), and therefore may alter DC-mediated antigen presentation. Given the complex protective and undesired immunomodulatory effects of both drugs, the timing and duration of their administration need to be appropriately adjusted and tailored to improve patient outcome according to clinical data obtained from recently completed and ongoing clinical trials.

Mechanistically, elevated levels of BTK activity have been reported in blood monocytes from patients with severe COVID-19 compared with those from healthy volunteers (138, 139, 141, 147, 148). *Btk* ^-/-^ monocytes, macrophages and neutrophils show defects in TLR- and NLRP3- induced NF-κB activation as well as impaired production of inflammatory cytokines and chemokines (24). Acalabrutinib and ibrutinib are able to inhibit monocyte, macrophage and neutrophil activation, and thus decrease the levels of inflammatory cytokines and chemokines such as IL-6, TNFα, IL-1, IFNγ and MCP-1 in patients with severe COVID-19 (**Figure 1**) (138–140, 142). In addition, ITK-dependent effects of ibrutinib on T cell differentiation, effector function and survival may also contribute to its modulatory effects on immunopathology and lymphopenia in COVID-19 therapy, skewing T cells from a T_H_2-dominant to a T_H_1 and CD8 cytotoxic populations and thus promoting a T_H_1 antiviral immunity (**Figure 1**) (24, 142, 143). However, two recently completed phase II trials of acalabrutinib (NCT04346199 and NCT04380688) in COVID-19 patients hospitalized with respiratory symptoms did not meet the primary efficacy endpoint of increasing the proportion of patients who remained alive and free of respiratory failure (142) (**Table 2**). Therefore, the exact efficacy of BTK inhibitors on COVID-19 remains to be clarified with additional clinical data from the recently completed and ongoing trials, and more mechanistic studies are needed to better understand how the drugs inhibit cytokine storm and improve lymphopenia in patients with severe COVID-19.

The promising clinical data of ibrutinib and acalabrutinib at inhibiting cytokine storm and improving lymphopenia in COVID-19 patients will likely promote further development of additional trials to expand the use of BTK inhibitors to sepsis and other infectious and inflammatory diseases. In particular, abundant preclinical evidence has demonstrated the efficacy of both ibrutinib and acalabrutinib at preventing and inhibiting cytokine storm as well as protecting against organ damage in several animal models of sepsis, including polymicrobial sepsis, cecal ligation and puncture (CLP)-induced sepsis, and LPS or LPS/galactosamine-induced sepsis (149–152). Evidence obtained from *Xid* mice with an inactivating mutation of *Btk* verified that the protective effects of BTK inhibitors in polymicrobial sepsis are mediated solely by inhibition of BTK (149). Mechanistically, BTK inhibitors suppresses the activation of TLR-NF-κB and NLRP3 inflammasomes in myeloid cells, leading to decreased release of cytokines and chemokines, reduced innate immune cell recruitment and a switch of macrophages from M1 to M2 phenotype (24, 149–151). In line with the animal evidence, increased BTK expression in blood cells is associated with poor survival in patients with sepsis (149). Together, all the above preclinical evidence supports the development of clinical trials to test ibrutinib and acalabrutinib for the treatment of sepsis, a major life-threatening health burden worldwide.

Interestingly, preclinical evidence also suggests potential application of ibrutinib and acalabrutinib in a number of other infectious and inflammatory diseases. This has been shown with animal models of *Mycobacterium tuberculosis* (*Mtb*) infection, visceral leishmaniasis caused by *Leishmania donovani*, *Influenza A* infection-induced acute lung injury, *Streptococcus pneumoniae*-induced acute pulmonary inflammation, high-fat-diet (HFD)-induced metabolic inflammation, endometriosis, post-ischemic brain inflammation after stroke and neuroinflammation-induced depression (26, 124, 153–159). On the other hand, CLL and MCL patients treated with BTK inhibitors exhibit increased risk of invasive fungal infection, bacterial infection and hepatitis B reactivation (32, 160–168). Relevant to these clinical observations, *Btk* ^-/-^ or ibrutinib/acalabrutinib-treated monocytes and macrophages exhibit defective TLR9-, TREM-1 and Dectin-1-mediated phagocytosis in response to fungal infections (24, 163, 169–172). In this scenario, clinical use of ibrutinib and acalabrutinib in infectious and inflammatory diseases requires prudent considerations of both the benefits and risks depending on the specific disease settings and patients’ immune cell and genetic contexts.

## Major Toxicities and Potential Limitations of Ibrutinib and Acalabrutinib in Therapeutic Use

Clinical data obtained from patients with CLL and MCL have shown that compared to conventional chemoimmunotherapy regimens, ibrutinib has a favorable safety profile and is generally well-tolerated (10, 17, 19, 20, 38). However, a unique set of toxicities has been reported for ibrutinib. Common adverse effects of ibrutinib observed in CLL and MCL patients include bleeding, atrial fibrillation, hypertension, neutropenia, arthralgias, myalgias, headache, diarrhea, nausea, fatigue, rash and infection (38, 162, 166, 173–180). Based on the preliminary results posted at ClinicalTrials.gov, commonly observed adverse effects of ibrutinib in patients with other human diseases include fatigue, anemia, gastrointestinal disorders, thrombocytopenia, myalgia, arthralgia, bleeding and infection (**Table 2**). These toxicities are mediated by both on-target inhibition of BTK and variable off-target inhibition of other kinases such as ITK, TEC, CSK, ERBB2/HER2, ERBB4/HER4, SRC, BMX, JAK3, EGFR, PTK6, c-Kit and PDGFR, etc. (38, 173, 178, 181, 182). Severe adverse events lead to discontinuation of ibrutinib treatment in 9 – 23% of CLL and MCL patients (38) and in occasional cases of other human diseases (ClinicalTrials.gov).

Because of its higher biochemical and cellular selectivity, acalabrutinib has an improved safety profile and exhibits a high efficacy in ibrutinib-intolerant CLL patients (6, 11, 12, 183–187). Despite its improved specificity and toxicity profile, common adverse effects of acalabrutinib have also been reported, including headache, diarrhea, fatigue, myalgias, cough, neutropenia, nausea, skin rash and infection (181, 186, 188–191) (**Table 2**). Severe adverse events of acalabrutinib are relatively rarer than those of ibrutinib in patients with B cell malignancies and other diseases (181, 186, 188–191) (ClinicalTrials.gov). These data suggest that acalabrutinib may be an option for ibrutinib-intolerant patients with B cell malignancies as well as other diseases.

Due to its off-target inhibition of ITK, TEC and other kinases, ibrutinib has stronger and broader immunomodulatory effects than acalabrutinib (24). Of clinical importance, one major difference between the immunomodulatory effects of these two BTK inhibitors is that ibrutinib, but not acalabrutinib, impairs FcγR-mediated antibody-dependent cellular cytotoxicity (ADCC) in human NK cells and antibody-dependent cellular phagocytosis (ADCP) in human macrophages and neutrophils (24, 35, 36, 192–198). In line with this notion, a recent phase Ib/II study (NCT02296918) showed that acalabrutinib in combination with obinutuzumab (an anti-CD20 with enhanced ADCC activity) produce high and durable responses that deepen over time in CLL patients, while ibrutinib plus rituximab or obinutuzumab do not show benefits over the respective monotherapies (199, 200). Therefore, ibrutinib might have relatively limited potential in combination therapies with a variety of antibodies used for the treatment of different human diseases whose mechanisms of action depend on ADCC or ADCP. When designing combination therapies involving ibrutinib and antibodies, appropriate sequential or alternate dosing schedules of ibrutinib *versus* antibody treatment episodes should be considered and would be more effective than concurrent administration of the drugs.

For both ibrutinib and acalabrutinib, one limitation is that after long-term treatment many patients acquire resistance caused by mutations of Cys481 in the kinase domain of BTK, the covalent binding site for both drugs (38). This limitation could be potentially overcome by the development of non-covalent binding BTK inhibitors such as fenebrutinib (GDC-0853), ARQ 531 (ArQule 531) or LOXO-305 (RXC005, REDDX08608) (38). Another potential common limitation for all BTK inhibitors is that long-term treatment with BTK inhibitors may affect bone homoeostasis and bone structure due to inhibition of osteoclast differentiation and function, as BTK is essential for RANKL-induced signaling pathways in osteoclasts (24, 113, 201, 202). These limitations suggest that combination therapies exploiting BTK inhibitors and other effective drugs with distinct mechanisms of activation and toxicity profiles, provided with appropriate dosing and treatment schedules, will help to enhance the efficacy while reducing the severity of adverse effects induced by each individual drug, thereby improving patient outcome.

## Summary

The BTK inhibitors ibrutinib and acalabrutinib are FDA-approved drugs for the treatment of B cell malignances and have demonstrated unprecedented success in CLL patients. Mounting preclinical and clinical evidence indicates that both ibrutinib and acalabrutinib are versatile and have direct effects on many immune cell subsets as well as other cell types beyond B cells. The versatility and immunomodulatory effects of both drugs have been exploited to expand their therapeutic potential in a wide variety of human diseases beyond B cell malignancies. Ibrutinib and acalabrutinib, as monotherapies or as part of combination therapies, are being tested in clinical trials in patients with hematological malignancies of myeloid cells and T cells, various solid tumors, cGVHD, autoimmune diseases, anaphylaxis and COVID-19. Clinical results obtained from these ongoing trials will provide valuable information to guide the design and improvement of future trials, including optimization of combination regimens and dosing sequences as well as better patient stratification and more efficient delivery strategies. Such information will further advance the precise implementation of ibrutinib and acalabrutinib into the clinical toolbox for the treatment of different human diseases.

## Author Contributions

PX, SZ, and SG have taken the leading roles in designing and writing this manuscript. JJ, EV, and JA have also made significant contributions to writing this manuscript, especially the section of COVID-19 and sepsis. All authors contributed to the article and approved the submitted version.

## Funding

This study was supported by a research grant from Acerta Pharma, the National Institutes of Health grants R01 CA158402 and R21 AI128264 (PX), the Department of Defense grant W81XWH-13-1-0242 (PX), a New Jersey Commission on Cancer Research (NJCCR) grant DCHS19CRF005 (PX), a Busch Biomedical Grant (PX) and a Pilot Award from the Cancer Institute of New Jersey through Grant Number P30CA072720 from the National Cancer Institute (PX). Acerta Pharma, LLC. was not involved in the study design, collection, analysis, interpretation of data, the writing of this article or the decision to submit it for publication.

## Conflict of Interest

The authors declare that the research was conducted in the absence of any commercial or financial relationships that could be construed as a potential conflict of interest.

## Publisher’s Note

All claims expressed in this article are solely those of the authors and do not necessarily represent those of their affiliated organizations, or those of the publisher, the editors and the reviewers. Any product that may be evaluated in this article, or claim that may be made by its manufacturer, is not guaranteed or endorsed by the publisher.
